# Differences in bacterial community structure and metabolites between the root zone soil of the new high – Fragrance tea variety Jinlong No. 4 and its grandparent Huangdan

**DOI:** 10.1371/journal.pone.0318659

**Published:** 2025-02-21

**Authors:** Jun Sun, Zhikun Lin, Liugang Zhu, Wenjin Zhang

**Affiliations:** 1 Tea Research Institute of Fujian Academy of Agricultural Sciences, Fuzhou, China; 2 Agricultural Sciences Research Institute of Fuzhou, Fuzhou, China; Universidade de Coimbra, PORTUGAL

## Abstract

With the development of the tea industry, understanding the differences in root zone microecology among different tea varieties is of great significance for improving tea quality and yield. To investigate the microbial and metabolite foundation underlying the disparities in root zone physicochemical properties between the high-aroma new tea variety JL4 (Jinlong No.4) and its grandparent HD (Huangdan), the bacterial diversity, community structure and metabolite distinctions of HD and JL4 root zone soils were analyzed using NovaSeq 6000 high-throughput sequencing and GC-MS-derived metabolomics technologies. The analysis of soil physical and chemical properties showed that, compared with HD, the available phosphorus (AP) in JL4 was significantly decreased (28.91 ±  9.78 mg · kg^−1^, *P* < 0.05), and so was the available potassium (AK) at 57.67 ±  4.04 mg · kg^−1^. The results from 16S rDNA sequencing indicated that, compared with HD, JL4 had a lower Shannon index and a higher abundance of Gram-negative and aerobic-related bacteria. These results indicated that there was a decrease in bacterial diversity in the root zone soil of JL4. The dominant bacterial phyla included *Proteobacteria*, *Acidobacteriota*, and *Chloroflexi* among others. Biomarkers in HD included *Firmicutes*, *Rhizobiales*, and *Caulobacterales*, and biomarkers for JL4 comprised *Sphingomonadaceae bacterium URHD0088* and *Halomonadaceae*. GC-MS derivatization metabolomics highlighted sugars as having the most differential metabolites (8). In JL4, D-manitol 2 and scylo-inositol decreased while (-)-epicatechin, catechin, and D-pinitol increased. KEGG pathway enrichment analysis revealed substantial enrichment in metabolic pathways related to flavonoid biosynthesis. The changes in these metabolites may have a significant impact on the growth and quality of tea plants. Redundancy Analysis (RDA) along with correlation analyses indicated significant impacts on root zone bacterial community structure by factors such as AK, Soil Organic Matter (SOM), NO_3_^−^-N (nitrate nitrogen), and pH levels. A significant positive correlation was observed between AK and both *Firmicutes* and *Kapabacteria* individually; furthermore, AP exhibited a highly significant positive correlation with *Kapabacteria* but a significant negative correlation with *unidentified Archaea*. Catechin and (-)-Epicatechin were significantly negatively correlated with *Actinobacteria* phylum while showing a significant positive correlation with *Verrucomicrobia* and *Kryptonia* phyla. This study systematically compared the microbial and metabolite characteristics of the root zone soil of JL4 and HD for the first time, providing new ideas and methods for tea variety improvement and precision cultivation management, which is expected to promote the high-quality development of the tea industry in the future.

## Introduction

Tea plant [*Camellia sinensis* (L.) O. Kuntze] is a globally cultivated cash crop [1]. The environmental conditions of the soil in which it grows have a critical impact on tea quality and yield [2,3]. In recent years, with the advancement of agricultural science and technology and the rising demand for tea quality from consumers, breeding and promoting new varieties with high-aroma has become an important development direction for the tea industry [4]. HD is a parent for breeding many new high-aroma tea varieties and is also a commonly used regional trial control specialty for our oolong tea varieties [5,6]. JL4 is a new high-aroma variety selected from the offspring of Jinguanyin, a descendant of HD. The oolong tea produced from it has a rich floral aroma, mellow taste, and excellent quality. As a new high-aroma tea variety, JL4 has drawn extensive attention due to its unique aroma components and outstanding quality. However, compared with traditional varieties, the physical and chemical properties of the root zone soil of new varieties may differ, potentially affecting the growth and development of tea plants.

The root zone soil, which includes the rhizosphere soil and the adjacent soil regions affected by plant roots to a certain extent, is a crucial domain in plant-soil interactions. The rhizosphere soil refers to the soil area around plant roots that is influenced by the roots [7]. In this area, plant roots not only absorb the necessary water, nutrients, and other substances from the soil, but also release some of the metabolites produced by the plant itself into the soil, thereby improving the soil microenvironment and contributing to the formation of its unique rhizosphere microbial community structure. Rhizosphere microorganisms are a type of microorganisms that directly affect the growth and development of plant roots within the soil environment. Tea plant rhizosphere soil bacteria, acting as a pivotal force in nutrient cycling within the microenvironment, play a crucial role in enhancing soil fertility and facilitating the normal growth of tea plants. Their multifaceted functions encompass secreting auxins, engaging in biological nitrogen fixation, accelerating the decomposition of soil organic matter, and mineralizing nutrients, all of which contribute to the overall health and vitality of the tea ecosystem. Tea plant rhizosphere soil metabolites encompass a diverse array of organic compounds secreted by both microorganisms and plant roots. These metabolites not only provide the necessary energy for rhizosphere microorganisms, but also directly affect the quantity and population structure of rhizosphere microorganisms. Research has demonstrated that distinct tea varieties can selectively enrich or exclude specific microbial populations due to variations in their root exudates. This, in turn, can significantly impact the physical and chemical properties as well as the metabolic processes within the rhizosphere soil. Consequently, investigating the microbial and metabolite underpinnings that drive differences in root zone soil properties between the novel high-aroma tea variety JL4 and its ancestral variety HD holds significant importance.

Previous studies on the rhizosphere soil of tea plants mainly focused on the relationship between tea quality and the soil environment, the structure and function of microbial communities, the development of microbial resources, and soil improvement and fertilization strategies [8–15]. However, there has been no comprehensive study on the root zone soil of specific high-aroma new tea varieties, and research on the physical and chemical properties and synergistic mechanism between microorganisms and metabolites still needs to be further strengthened.

In this study, high-throughput sequencing technology and GC-MS-derived metabonomics technology were employed to comprehensively detect the metabolites in the root zone soil of two tea varieties and identify the key metabolites related to changes in soil physical and chemical properties. Based on the microbial community structure and metabolite composition data, statistical analysis methods were used to discuss the correlation between microbial communities and metabolites and reveal their synergistic effects on soil physical and chemical properties.

The objective of this study was to investigate the microbial and metabolite underpinnings responsible for the disparities in the physicochemical properties of root zone soil between the novel high-aroma tea variety JL4 and HD. Through a comparative analysis of the root zone soil characteristics of these two tea varieties, we sought to uncover the key factors influencing soil quality and tea growth. This research offers a fresh perspective and theoretical foundation for elucidating the distinctions in root zone soil environments between high-aroma tea varieties and conventional varieties. Furthermore, it provides scientific guidance for tailoring soil conditions to the specific needs of different tea varieties, thereby enhancing tea quality and yield, and fostering high-quality tea cultivation and soil health management. Additionally, this study aids in reducing the reliance on chemical fertilizers and pesticides, advancing sustainable tea production, and safeguarding the environment. Moreover, it contributes to a more profound understanding of the intricate tea root zone soil ecosystem, promotes deeper insights into the mechanisms of soil-plant-microbial interactions, enriches the theoretical knowledge of soil ecology and plant nutrition, and furthers the sustainable development of agriculture and ecological environmental protection.

## Materials and methods

### Experimental design, soil sampling and preparation

The experimental site is located within the Tea Research Institute of Fujian Academy of Agricultural Sciences (in Shekou Town, Fu’an City, Fujian Province, China (119°57’E, 27°22’N)). It is the research facility owned by this institution, and the researchers of this institution do not need additional site-access permits. Characterized by a subtropical marine monsoon climate, the site has an altitude of 91 meters, with an average annual temperature ranging from 13.4 °C to 20.2 °C and an average annual precipitation ranging from 1250 mm to 2350 mm. The frost-free period lasts for 235 to 300 days. A total of six tea varieties, including the experimental variety JL4, the control variety HD, Shuixian and three other descendants of Jinguanyin, were planted in a random block arrangement with three plots in April 2016. Protective rows were established around these plants. The adopted planting method was single-row, double-plant strip replanting, maintaining a plant spacing of 33 cm and a row spacing of 150 cm. All tea trees were cultivated and managed in accordance with conventional tea tree management practices.

In April 2023, when the tea trees had matured to 7 years of age, five tea trees were randomly sampled from each treatment group. Soil from the root area, at a depth of 0–20 cm, was collected within a 10 cm diameter circle around each tree, after meticulously removing the topsoil. The collected five samples were then mixed into a single composite sample, and impurities were removed. Each sample collected from a separate plot for each variety was considered as one replicate, resulting in a total of three replicates per variety. Following this, the composite soil sample was divided into three equal parts. The first part underwent air-drying and sieving procedures, preparing it for the determination of soil physicochemical properties. The second part was stored in a −80 °C ultra-low temperature refrigerator, reserved specifically for high-throughput MiSeq sequencing of the soil microbial community. The third part was also stored in a −80 °C refrigerator, intended for GC-MS derivatization and subsequent metabolite detection.

### Analysis of physical and chemical properties

The soil pH was measured using an E20-FiveEasy pH meter (Mettler Toledo, Germany), while the soil electrical conductivity (EC) was determined with an electric conductometer. For soil measurements, a soil-water suspension was prepared (2.5:1 mixture of deionized water and fresh soil for pH, and 5:1 for EC) and shaken for 30 min. Nitrate (NO_3_^−^-N) and ammonium (NH_4_^+^-N) were extracted by adding 5 g of fresh soil to 50 ml of a 2 M KCl solution. After shaking for 1 h and allowing it to stand for another hour, the supernatant was filtered through glass fiber filters (Fisher G4, 1.2-μm pore space). The concentrations of NO_3_^−^-N and NH_4_^+^-N were then determined using a continuous-flow analytical system (San++ system; Skalar, Holland). Available phosphorus (P) was extracted with a 0.5 M NaHCO_3_ solution and measured by the Mo-Sb colorimetric method. Available potassium (K) was extracted with 1 M ammonium acetate (NH_4_OAc) and measured by the flame spectrophotometry method. Soil organic matter (SOM) was measured using the K_2_Cr_2_O_7_-H_2_SO_4_ oxidation method. The soil particle size was determined using a laser particle size analyzer (Bettersize 3000, Baite Dandong Instrument Co., Ltd., China) with a measurement range of 0.02-2000 μm. The particle size standard followed the international sediment particle size classification, which included sand particles >20 μm, silt particles 2–20 μm, and clay particles <2 μm.

### Soil metabolite analysis

Fresh soil was collected, weighed accurately, promptly frozen in liquid nitrogen, and stored at −80 °C until utilization. Samples were freeze-dried and subsequently ground into powder at room temperature. Weigh 0.5 g of the sample, add 1 mL of methanol: isopropanol: water (3:3:2 V/V/V) extract, vortex for 3 min and subject to ultrasound for 20 min. The extracts were centrifuged at 12000 r/min at 4°C for 3 min. The supernatant was carefully transferred into a sample vial and 0.020 mL of internal standard (10 μg/mL) was added to evaporate under nitrogen flow. The evaporated samples were transferred to the lyophilizer for freeze-drying. The residue was utilized for further derivatization.

The derivatization method was as follows: The sample was mixed with 0.1 mL of a solution of methoxyamine hydrochloride in pyridine (0.015 g/mL). The mixture was incubated at 37 °C for 2 h. Then 0.1 mL of BSTFA (with 1% TMCS) was added to the mixture and kept at 37 °C for 30 min after vortex-mixing. 0.2 mL of the derivatization solution was pipetted, n-hexane was added to dilute to 1 mL, filtered through a 0.22 μm organic phase syringe filter, stored in a refrigerator at −20 °C, and tested on the machine within 24 hours.

Agilent 8890 gas chromatograph (Santa Clara, CA) coupled to a 5977B mass spectrometer with a DB-5MS column (30 m length ×  0.25 mm i.d. ×  0.25 μm film thickness, J&W Scientific, USA) was utilized for GC-MS analysis of the extracting solution. Helium was employed as the carrier gas, at a flow rate of 1.2 mL/min. Injections were made in the front inlet mode with a split ratio of 5:1, and the injection volume was 1 μL. The oven temperature was maintained at 40 °C for 1 min, and then raised to 100 °C at 20 °C/min, raised to 300 °C at 15 °C/min, and held at 300 °C for 5 min. All samples were analyzed in scan mode. The ion source and transfer line temperature were 230 °C and 280 °C, respectively.

Data pretreatments including peak filtering, alignment, identification and normalization were conducted by Agilent MassHunter software. The Standard Database with PubChem (https://pubchem.ncbi.nlm.nih.gov/compound/), Chem960 (https://www.chem960.com/cas/) and ClassyFire (http://classyfire.wishartlab.com/#structure-query) [16] were utilized for structure identification. Variable Importance in Projection (VIP) and Fold Change (FC) values were calculated. Among these metabolites, VIP >  1.0, FC >  1.2 or FC <  0.8 were used as the selection criteria [17,18]. Orthogonal Partial Least Squares-Discriminant Analysis (OPLS-DA) was conducted by MetaboAnalystR 1.0.1 package in R v.3.5.1 [19]. Volcano plot, Scatter plot and Correlation chord plot were conducted by Pandas 0.23.4 package in Python 3.6.6 [20], ggplot2 3.3.0 package in R v.3.5.1 [21], ggplot2 3.4.0 package in R v.4.2.2 [21], and stats 3.5.1 package in R v.3.5.1 [22], respectively. Identified metabolites were annotated using KEGG Compound database (http://www.kegg.jp/kegg/compound/), annotated metabolites were then mapped to KEGG Pathway database (http://www.kegg.jp/kegg/pathway.html).

### Soil high-throughput sequencing

The total DNA in the soil was extracted using the CTAB method [23], and the purity and concentration of the extracted DNA were detected using 1% agarose gel electrophoresis. PCR primers 341F (5´- CCTAYGGGRBGCASCAG-3´) and 806R (5´- GACTACNNGGGTATCTAAT-3´) were used to amplify the V3-V4 region of bacterial 16S. The PCR product was purified by 2% agarose gel electrophoresis and recovered using a gel recovery kit provided by the Qiagen company. The TruSeq® DNA PCR Free Sample Preparation Kit was used for library construction, and the constructed library was subjected to quantitative quality inspection using a Qubit/Agilent Bioanalyzer 2100System/Q-PCR, followed by sequencing using NovaSeq 6000.

Quality control, splicing, and chimeric filtering were performed on the data obtained from the Illumina NovaSeq sequencing to obtain effective tags. The RDP classifier Bayesian algorithm (97% similarity level) was used for OTU (Operational Taxonomic Units) clustering. Subsequently, species annotation was performed on the representative sequences of each OTU, using photoseq v.1.40.0 and vegan v.2.6.2 packages in R v.4.2.0 to calculate the Chao 1, Shannon, Simpson, ace, Goods coverage, PD_whole_tree indices. Venn diagram analysis was conducted using the stats 3.5.1 package in R v.4.2.0. Principal Component Analysis (PCA) was conducted using stats 3.5.1 package in R v.3.5.1. LEfSe analysis (LEfSe v.1.1.2) was used to screen for differentially abundant bacteria in the root zone soil of different varieties with LDA >3.5. BugBase analysis (BugBase v.0.1.0) was conducted for phenotypic prediction analysis of bacterial communities [24]. FastSpar correlation analysis (FastSpar v1.0.0) was conducted to calculate the correlations between top 100 bacterial genera and, correlations with | r |  >0.8 and abundance ≥0.005% were selected. Correlation analysis between soil metabolites and bacteria was conducted using WGCNA 1.69 and corrplot 0.92 packages in R v.3.5.1 and R v.4.1.2. Redundancy analysis (RDA) and Pearson correlation analysis between soil physicochemical properties and bacterial communities were conducted using the vegan 2.5.6 package in R v.3.5.1 [25] and ComplexHeatmap package in R.

## Results

### Physical and chemical properties of root zone soil

The physical and chemical properties of the root zone soils from JL4 and HD were analyzed, and the results are presented in Table 1. In comparison to HD, the JL4 root zone soil exhibited increases in clay (<2 μm) (7.00 ±  0.59%), silt (2–20 μm) (38.63 ±  0.81%), and NO_3_^−^-N (3.38 ±  1.32 mg · kg^−1^). Conversely, the concentrations of AP (28.91 ±  9.78 mg · kg^−1^), AK (57.67 ±  4.04 mg · kg^−1^), SOM (1.35 ±  0.03 g · kg^−1^), NH_4_⁺ -N (9.12 ±  1.06 mg · kg^−1^), as well as pH (4.44 ±  0.16) and EC (53.20 ±  12.5 μS/cm) all showed a decrease in JL4 compared with HD. Notably, significant differences were observed in the contents of AP and AK, with *P* <  0.05 indicating statistical significance.

**Table 1:** The physical and chemical properties of root zone soil.

Item	HD	JL4
AP (mg · kg^−1^)	82.62 ± 30.34 a	28.91 ± 9.78 b
AK (mg · kg^−1^)	97.67 ± 29.26 a	57.67 ± 4.04 b
SOM (g · kg^−1^)	1.65 ± 0.20 a	1.35 ± 0.03 a
NH_4_+-N (mg · kg^−1^)	9.58 ± 0.74 a	9.12 ± 1.06 a
NO_3_^−^-N (mg · kg^−1^)	2.53 ± 1.07 a	3.38 ± 1.32 a
pH	4.50 ± 0.09 a	4.44 ± 0.16 a
EC (μS/cm)	63.6 ± 2.26 a	53.20 ± 12.5 a
Clay (<2 μm) (%)	5.82 ± 1.34 a	7.00 ± 0.59 a
Silt (2–20 μm) (%)	34.66 ± 7.65 a	38.63 ± 0.81 a
Sand (>20 μm) (%)	59.52 ± 8.98 a	54.37 ± 1.34 a

SOM: organic matter. NO_3_^−^-N: nitrate nitrogen. NH_4_^+^-N: ammonium nitrogen. AK: available potassium. AP: available phosphorus. EC: electrical conductivity. Sand (>20 μm): the proportion or amount of sand particles with diameter greater than 20 μm. Silt (2–20 μm): the proportion or amount of silt particles with diameter between 2 and 20 μm. Clay (<2 μm): the proportion or amount of clay particles with diameter less than 2 μm. Different letters in the same row indicate statistical significance (*P* <  0.05).

### Metabolites in the root zone soil of different tea varieties different metabolites

**Fig 1:**
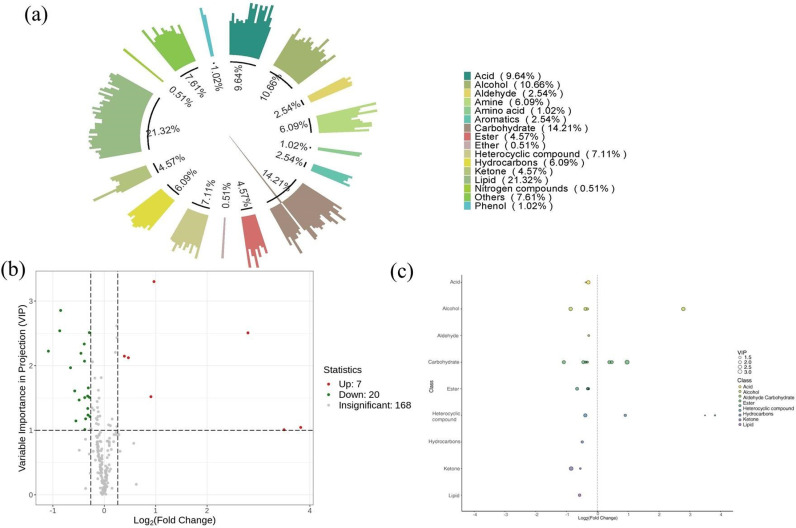
Detected metabolites (a), volcano plot (b) and scatter plot (c) of differential metabolites.

A total of 296 metabolites, including amino acids, hydrocarbons, carbohydrates, ketones, lipids, alcohols, etc., were detected and identified among all soil samples (Fig 1). Of these compounds, the number of lipids was the greatest, accounting to 21.32% of the whole metabolites, followed by carbohydrates (14.21%), alcohols (10.66%), and acid (9.64%). VIP and FC values were calculated, and metabolites with VIP >  1.0, FC >  1.2 or FC <  0.8 were selected as criteria. A total of 20 metabolites were significantly decreased, and 7 were significantly increased. These differential metabolites included alcohols, aldehydes, acids, sugars, hydrocarbons, ketones, heterocyclic compounds, lipids, esters, etc. Among them, sugars (8) are the most abundant, followed by alcohols (4) and heteroc

### Correlation analysis of differential metabolites in root zone soil of different tea varieties

**Fig 2:**
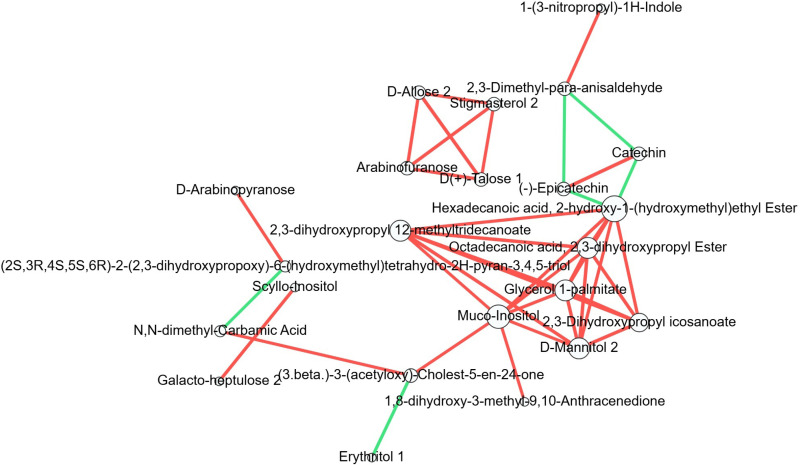
Correlation of differential metabolites. Circles represent distinct differential metabolites, and the larger the number of metabolites associated with them, the larger the circle. The red lines represent positive correlations, and the green lines represent negative correlations. The thickness of the line represents the absolute value of the Pearson correlation coefficient r (the thicker the line, the larger the | r|).

To further understand the mutual regulatory relationship among metabolites of different varieties, correlation analysis (Pearson) was performed on the identified differential metabolites, and correlations with *P* < 0.05 and | r | >  0.8 were shown in Fig 2. Among them, 2,3-dihydroxypropyl icosanoate, D-mannitol 2, glycerol 1-palmate, and octadecanoic acid, 2,3-dihydroxypropyl ester were involved in the most correlations. Moreover, they were positively correlated with each other. Hexadecanoic acid, 2-hydroxy-1- (hydroxymethyl) ethyl ester and 2,3-dihydroxypropyl 12-methyldecanoate were next, which were positively correlated with other five differential metabolites. Interestingly(-) epicatechin and catechin were positively correlated with the highest correlation coefficient. In addition, D (+) - talose 1, arabinofuranose, D-allose 2, and stigmasterol 2 were positively correlated. However(3β)-3-(acetyloxy)-cholest-5-en-24-one was negatively correlated with erythritol 1, and (2S, 3R, 4S, 5S, 6R)-2-(2,3-dihydroxypropargy)-6-(hydroxymethyl) tetrahydro-2H-pyran-3,4,5-triol was negatively correlated with N,N-dimethyl-carbamic acid. Carbohydrate of D (+) - talose 1, arabinofuranose and D-allose 2, and alcohol of stigmasterol 2 were all decreased. They were positively correlated with each other.

### KEGG functional annotation and enrichment analysis of differential metabolite

**Fig 3:**
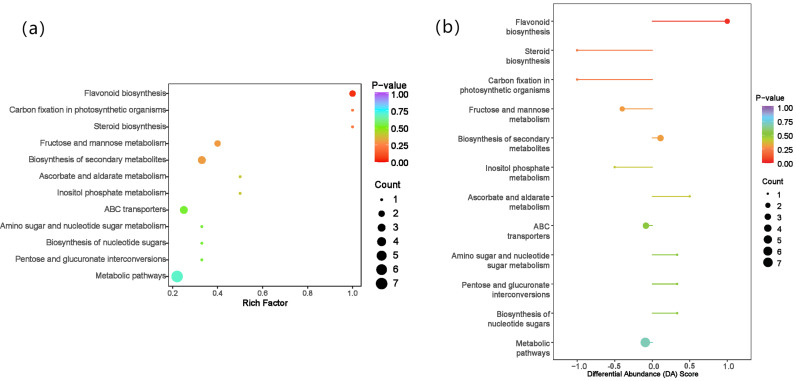
KEGG enrichment map of differential metabolites (a) and differential abundance score map (b).

Annotation of KEGG metabolites with significant differences among different tea varieties was conducted, and KEGG pathway enrichment analysis was performed. The results showed that the metabolic pathways of flavonoid biosynthesis, carbon fixation in photosynthetic organisms, and steroid biosynthesis were significantly and highly enriched (Fig 3). The differential abundance score (DA Score) analysis showed that the expression trend of flavonoid biosynthesis metabolites was increased (DA Score =  1, *P* =  0.04).

### Bacterial community structure and diversity in root zone soil of different tea varieties

**Fig 4:**
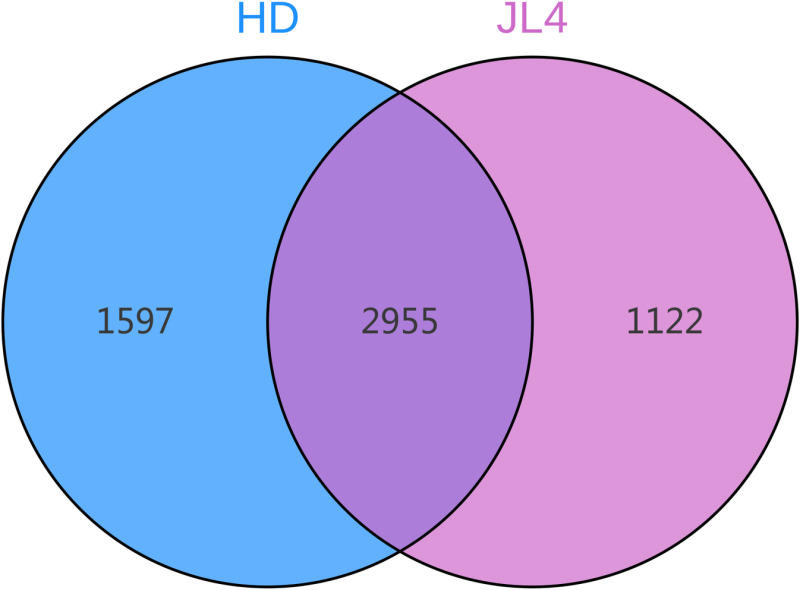
OUT Venn diagram.

The Venn plot of root zone soil of different tea tree varieties (Fig 4) showed that there were 2919 OTUs shared by HD and JL4. JL4 had fewer unique OTUs than HD. Among them, JL4 had 1141 unique OTUs and HD had 1603 unique OTUs.

**Fig 5:**
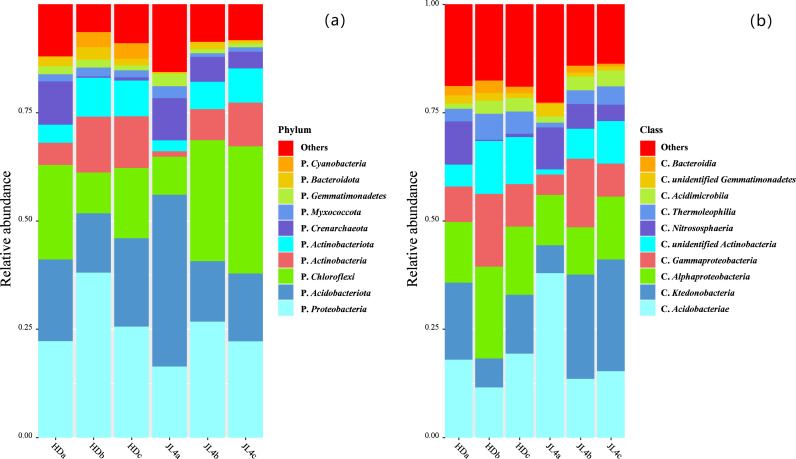
Relative abundance of bacteria phyla (a) and classes (b) in root zone soil of different tea varieties. C, means the class level; P, means the phylum level.

Screening representative OTU sequences at a 97% similarity level for taxonomic analysis using the RDP classifier with the Bayesian algorithm, the results (Fig 5) showed that at the phylum level, the dominant bacterial groups were *Cyanobacteria, Bacteroidota, Gemmatimonadetes*, *Myxococcota*, *Crenarchaeota*, *Actinobacteriota*, *Actinobacteria*, *Chloroflexi*, *Acidobacteriota*, and *Proteobacteria*. Among them, *Proteobacteria*, *Actinobacteriota*, *Actinobacteria*, *Bacteroidota*, and *Cyanobacteria* had relatively high abundances in the HD root zone soil, while *Crenarchaeota*, *Chloroflexi*, *and Acidobacteriota* had relatively high abundances in the JL4 root zone soil.

The dominant microbial communities at the class level were *Bacteroidia*, *unidentified Gemmatimonadetes*, *Acidimicrobia*, *Thermophilia*, *Nitrosophaeria*, *unidentified Actinobacteria*, *Gammaproteobacteria*, *Alphaproteobacteria*, *Ktedonobacteria*, and *Acidobacteriae*. Among them, *Acidobacteriae*, *Ktedonobacteria*, and *Nitrosophaeria* had relatively high abundances in the JL4 root zone soil, while *Unidentified Actinobacteria*, *Gammaproteobacteria, Alphaproteobacteria*, *Thermophilia*, and *Bacteroidia* had relatively high abundances in the HD root zone soil.

The soil bacterial community richness and diversity results were presented in Table 2. Simpson’s index of diversity (1 – D) of root zone soil bacteria in both varieties was 0.99. The indices of OTUs, Shannon, Chao1, Ace, and PD_whole_tree of HD were higher than those of JL4. Among them, a significant difference was found in the Shannon index between the two varieties (*P* < 0.05).

**Table 2:** The soil bacterial community richness and diversity in different tea varieties root zone soil.

Item	HD	JL4
OUTs	2601 ± 604.47 a	2338.67 ± 351.2 a
Shannon	9.05 ± 0.14 a	8.48 ± 0.27 b
Simpson	0.99 ± 0 a	0.99 ± 0 a
Chao1	2949.36 ± 800.35 a	2721.48 ± 395.14 a
Ace	3015.37 ± 867.33 a	2813.9 ± 464.59 a
Goods coverage	0.99 ± 0 a	0.99 ± 0 a
PD_whole_tree	207.53 ± 57.49 a	192.89 ± 7.9 a

Different letters in the same row indicate statistical significance (*P* <  0.05).

**Fig 6:**
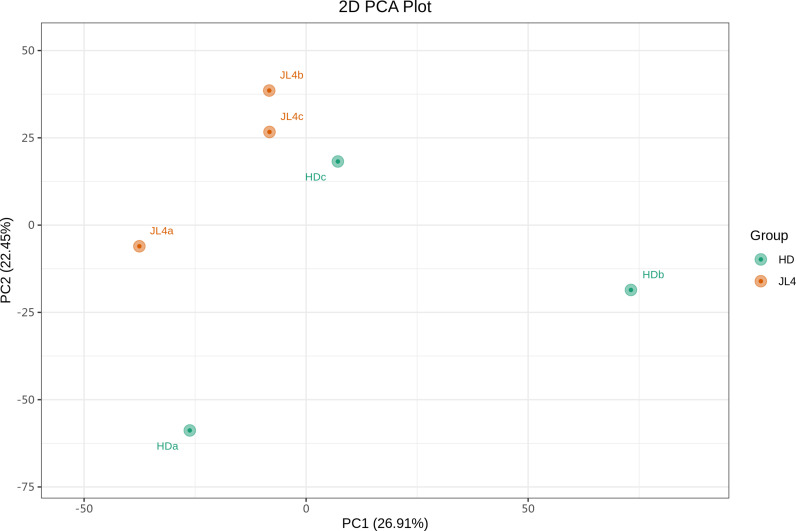
PCA of bacterial communities.

To explore the differences in the structure of root zone soil bacterial communities among different tea varieties, PCA was conducted on the soil bacterial communities. The results showed (Fig 6) that the contribution rates of PC1 and PC2 were 26.91% and 22.45%, respectively, accounting for a total of 49.36%. The two tea varieties could be slightly clustered together, while different varieties could be well distinguished. JL4 was distributed in the upper left corner relative to HD, indicating a certain difference in the root zone soil bacterial community between JL4 and HD.

**Fig 7:**
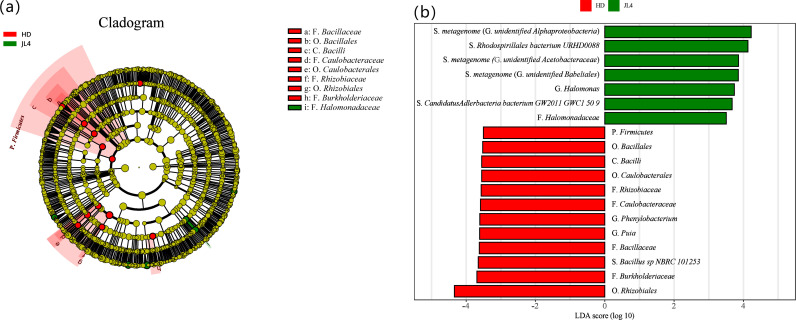
LEfSe analysis of root zone soil bacterium from different tea varieties. (a) LEf Se analysis evolutionary branching diagram. (b) LDA value distribution map. P, bacterial phylum; C, class; O, order; F, family; G, genus; S, species.

LEfSe analysis was used to screen for differentially bacteria in the root zone soil of the different varieties (*P* < 0.05). The results (shown in Fig 7) indicated that the biomarkers in HD included Order *Rhizobiales*, Family *Burkholderiaceae*, Species *Bacillus sp_NBRC_101253*, Family *Bacillaceae*, Genus *Puia*, Genus *Phenylobacterium*, Family *Caulobacteraceae*, Family *Rhizobiaceae*, Order *Caulobacterales*, Phylum *Firmicutes*, Class *Bacilli*, and Order *Bacillales*. The biomarkers in JL4 were Species *metagenome*, Species *Rhodospirillales_bacterium_URHD0088*, Genus *Halomonas*, Species *Candidatus Adlerbacteria bacterium GW2011 GWC1 50 9*, and Family *Halomonadaceae*. The above biomarkers played an important role in differentiating the community structure compositions of the root zone soils of different tea varieties.

**Fig 8:**
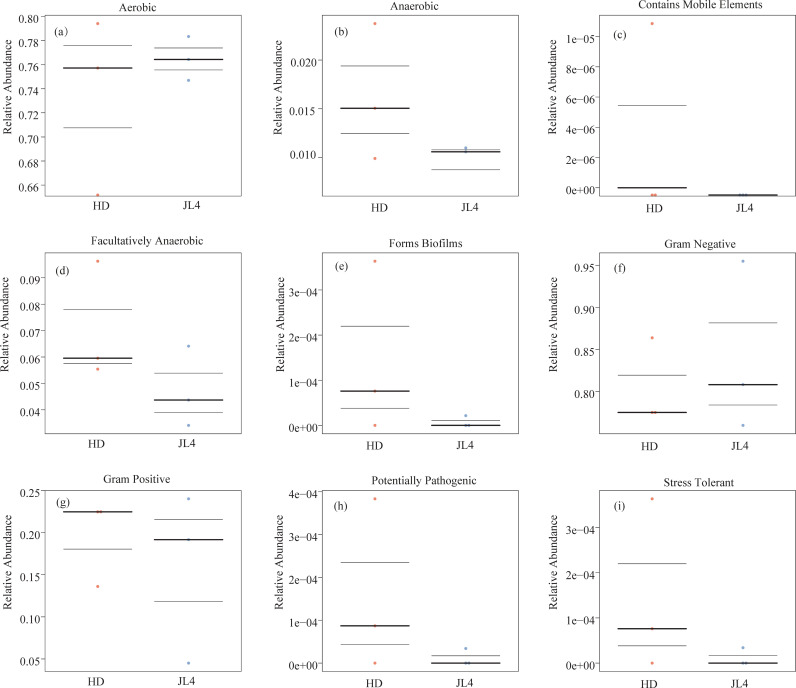
Prediction of bacterial BugBase phenotype. The three lines from bottom to top represent the lower quartile, mean, and upper quartile, respectively.

The bacterial community phenotype prediction analysis was performed using BugBase analysis. The abundance ratios and differences of bacteria with different phenotypes in different samples are shown in Fig 8. The relative abundances of Gram-negative bacteria and aerobic bacteria in JL4 were higher than those in HD, respectively. The relative abundances of other phenotypes such as anaerobic bacteria and bacteria containing mobile elements in JL4 were significantly lower than those in HD, respectively.

### Correlation between bacterial community structure and physicochemical properties in root zone soil of different tea varieties

**Fig 9:**
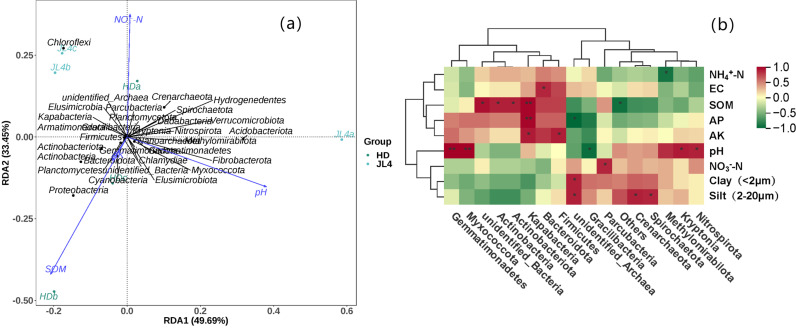
RDA and pearson correlation analysis of soil bacterial communities and soil environmental factors. (a) RDA. (b) Pearson correlation analysis. SOM: organic matter. NO_3_^−^-N: nitrate nitrogen. NH_4_^+^-N: ammonium nitrogen. AK: available potassium. AP: available phosphorus. EC: electrical conductivity. Sand (>20 μm): the proportion or amount of sand particles with diameter greater than 20 μm. Silt (2–20 μm): the proportion or amount of silt particles with diameter between 2 and 20 μm. Clay (<2 μm): the proportion or amount of clay particles with diameter less than 2 μm.

The redundancy analysis (RDA) between soil physicochemical properties and bacterial communities (Fig 9) showed that the physicochemical indicators significantly correlated with bacterial communities (with VIF < 10) were AK, SOM, NO_3_^−^-N, and pH. RDA1 and RDA2 accounted for 49.69% and 33.45%, respectively. Altogether, they accounted for 83.14% of the total variation in bacterial communities in the root zone soil of tea plants. Pearson correlation analysis with dominant bacterial phyla showed that SOM was significantly positively correlated with P. *Actinobacteria* and P. *Actinobacteriota*. It was also highly significantly positively correlated with P. *Kapabacteria*. pH was significantly positively correlated with P. *Gracilibacteria*, P. *Kryptonia*, and P. *Nitrospirota*, and highly significantly positively correlated with P. *Gemmatimonadetes* and P. *Myxococcota*. AP was highly significantly positively correlated with P. *Kapabacteria*. AK was significantly positively correlated with P. *Firmicutes* and P. *Kapabacteria*. EC was significantly positively correlated with P. *Bacteroidota*. NO_3_^−^-N was significantly positively correlated with P. *Parcubacteria*. NH_4_^+^-N was significantly positively correlated with P. *Methylomirabilota*.

## Discussion

As the genetically improved offspring of HD, JL4 retains the genetic blueprint of HD within its genetic makeup, yet it has undergone extensive gene recombination and optimization through the rigorous process of artificial selection and breeding. This inherent genetic distinction may subtly alter the composition and functionality of the microbial community residing in the root zone soil of both JL4 and HD tea plants.

In the root zone soil of both varieties, *Actinobacteria*, *Chloroflexi*, *Acidobacteria*, and *Proteobacteria* emerged as the predominant bacterial phyla, a finding that aligns with the established patterns observed in the rhizospheres of other tea cultivars [26–29]. However, a notable disparity was observed in the Shannon index, with JL4 exhibiting a significantly lower value (8.48 ±  0.27) compared to HD (9.05 ±  0.14), suggesting a reduction in the α diversity of root zone soil bacteria in JL4. To mitigate this and potentially enhance the bacterial diversity, tea cultivators can explore strategies such as the judicious application of organic fertilizer and the implementation of other cultivation and management practices [30,31].

Biomarkers for HD include F*. Burkholderiaceae*, F. *Rhizobiaceae, and* F. *Bacillaceae*, etc. In contrast, JL4 has biomarkers like G. *Halomonas*, S. *Candida adlerbacteria gw2011 gwc1509*, F. *Halomonadaceae*, etc. *Burkholderiaceae* form nodules in legume roots, converting atmospheric N_2_ to plant-available NH_3_. They can enhance plant growth activity, augment the microbial abundance in the vicinity of plant roots, effectively mitigate the occurrence of soil diseases, and certain strains of bacteria possess the capability to hydrolyze phosphorus. Additionally, these bacteria are positively correlated with biomass growth, suggesting their potential as plant growth-promoting rhizobacteria [32]. Microorganisms from *Rhizobia* family, *Burkholderiaceae*, and *Rhizobiaceae* are critical for legumes as they can form symbioses. The abundance of Gram-negative and aerobic bacteria in JL4 was conspicuously higher. Specifically, *Halomonadaceae* and *Halomonas*, both classified as Gram-negative bacteria, emerged as biomarkers in JL4. Despite the insignificant salinity difference in soils, other elements may account for halotolerant bacteria as cultivar markers. Different tea variety root exudates, like specific organic acids or secondary metabolites, could supply nutrients or signaling molecules that promote halotolerant bacteria growth and prevalence. Such substances may modify the bacteria’s metabolic and competitive capabilities in the soil microbial community.

Soil metabolites act as mediators of interaction between tea trees and soil microorganisms and play a crucial role in altering soil physicochemical properties. The AP and AK of JL4 root zone soil are significantly lower than those of HD, suggesting that soil AP and AK may be the main factors influencing the diversity of root zone soil bacterial communities in these two tea varieties. The difference indexes of soil physical and chemical properties vary among different tea varieties [29]. The most differential metabolites between the two plants are sugars, followed by alcohols and heterocyclic compounds. Arabinofuranose, along with other polysaccharides, exhibits a close association with the decomposition and transformation processes of plant residues and other organic materials within the soil. A proper quantity of D-Mannitol 2 is capable of enhancing the soil structure, augmenting the soil’s porosity and water-holding capacity. This, in turn, promotes the growth of plant roots and facilitates the absorption of essential nutrients, thereby indirectly augmenting the fertility of the soil. Erythritol has the potential to markedly decrease the dry weight of both the root and aerial parts of tomato plants. Additionally, it can impede the germination of corn and tomato seeds. These effects may consequentially and indirectly modify the structure and function of the soil microbial community in the surrounding environment [33]. D-Pinitol, a prevalent sugar alcohol in legumes, witnesses an upregulated biosynthesis in soybeans under soil drought conditions. The induced transcriptional activity of relevant genes prompts a significant accumulation of D-Pinitol in various plant organs, endowing the plants with enhanced tolerance to adversities such as drought [34]. Muco-Inositol and associated molecules in plants bolster tolerance against salt stress. They achieve this by safeguarding cell structures from reactive oxidants and regulating intracellular water pressure, consequently influencing plant growth in saline soils. Compared to HD, the expression trend of flavonoid biosynthesis metabolites in JL4 was increased (DA Score =  1, *P* =  0.04). This pathway is a part of the phenylpropanoid synthesis pathway. Moreover, flavonoids are widely distributed in plants and have diverse biological functions and roles in plant-environment interaction. They protect plants from UV radiation and are important in sexual reproduction. Flavonoids possess medicinal properties such as antiviral, antimutagenic, antilipoperoxidant, radioprotective, anti-complementary, anti-inflammatory, anti-tumor, antioxidant, and anti-inflammatory activities. Flavonoids, especially flavan 3-ols like (-)-epigallocatechin(-)-epicatechin(+)-gallocatechin(+)-catechin, and their gallate esters, are prominent metabolites due to their high content. Approximately 20% flavan 3-ols in tea is substantial. Tea’s health effects related to flavan 3-ols are based on antioxidative, anticancerogenic, antiallergenic, anti-inflammatory, and vasodilatory properties [35].

There are complex interactions among microbial communities, metabolites, and the physicochemical properties of soil. In our investigation, factors such as soil SOM, NO₃-N, pH, and AK have a substantial influence on the bacterial community structure within the root zone soil, which is consistent with the research results of Kong et al. [29]. Moreover, AK showed a significant positive correlation with bacterial phyla like *Firmicutes* and *Kapabacteria*, while AP had a notable positive correlation with *Kapabacteria*. However, Kong et al. [29] believed that *Firmicutes* were significantly positively correlated with root zone soil AP content. The inconsistency with the results of this study may be due to the different tea varieties studied.

Although this study has made certain progress in uncovering the differences in microbial communities and metabolites in the root zone soil between JL4 and HD, there are still some limitations. For example, this study primarily concentrated on the microbial communities and metabolites of root zone soil. However, it failed to fully consider the physiological and ecological characteristics of the aboveground parts of tea plants and their interaction with root zone soils. Future research can further combine the physiological and ecological processes of tea plant growth and development to further explore the interaction mechanism between microorganisms, plants, and soil. In addition, field experiments can be carried out to confirm the reproducibility and practicability of laboratory research results, providing a scientific basis for tea cultivation management and tea quality improvement. At the same time, with the continuous development of high-throughput sequencing technology and metabolomics technology, future studies are expected to reveal the complexity and diversity of microbial communities and metabolites in the root zone soil of tea plants.

## Conclusions

Based on NovaSeq 6000 high-throughput sequencing technology, the bacterial diversity and community structure of HD and JL4 root zone soils were identified, and the differences in root zone soil metabolites were identified by GC-MS-derived metabolomics technology. The analysis of soil physical and chemical properties showed that compared with HD, the AP (28.91 ±  9.78 mg · kg^−1^) and AK (57.67 ±  4.04 mg · kg^−1^) of JL4 were significantly reduced (*P* < 0.05). The results of 16S rDNA showed that the dominant bacterial phyla were *Proteobacteria*, *Acidobacteriota*, *Chloroflexi* and so on. The Shannon index of JL4 was significantly lower than that of HD. BugBase phenotype prediction analysis showed that the abundance of Gram negative and Aerobic related bacteria in JL4 was higher than that in HD, while other related bacteria such as Anaerobic were significantly lower than those in HD. LEfSe analysis showed that the Biomarkers in HD were P. *Firmicutes*, O. *Rhizobiales*, O. *Caulobacteras* et al. The Biomarkers in JL4 were S. *Rhodospirillales bacterium URHD0088*, F. *Halomonadaceae* et al. RDA and correlation analysis indicated that AK, SOM, NO₃⁻ - N, and pH had a significant impact on the structure of root zone soil bacterial communities. AK and P. *Firmicutes*, P. *Kapabacteria* were significantly positively correlated, individually. AP and P. *Kapabacteria* had a highly significant positive correlation. While AP and P. *Unidentified Archaea* were significantly negatively correlated. GC-MS derivatization metabolomics showed that among the differential metabolites, sugars (8 in number) had the most, followed by alcohols (4 in number) and heterocyclic compounds (4 in number). In JL4, D-mannitol 2 and scylo-Inositol decreased, while (-)-epicatechin, catechin, and D-pinitol increased. KEGG pathway enrichment analysis showed that the metabolic pathways of flavonoid biosynthesis was highly enriched. The bacteria, metabolites, and physicochemical factors in the root zone soil of tea plants above effected the root zone microecology of different tea varieties, providing a theoretical basis for improving the root zone microenvironment of tea plants and data support for the rational planting and distribution areas of tea varieties, as well as the breeding of tea varieties.

## Supporting information

S1 Data Alpha diversity, differential Metabolites, KEG DA score, lefse, Metabolites (all), OTUs, PCA components, RDA result.VIF and result. RDA. envfit. (ZIP)
